# Superselective Embolization of Arterial Bleeding as a Late Complication 3 Months After Nephron Sparing Surgery for Renal Cell Carcinoma

**DOI:** 10.1100/tsw.2004.88

**Published:** 2004-06-07

**Authors:** J. Thomas, F.G.E. Perabo, R. Bachmann, G. Steiner, H. Schild, S.C. Müller

**Affiliations:** Department of Urology, Rheinische Friedrich-Wilhelms-University of Bonn, Sigmund-Freud-Str. 25, 53105 Bonn, Germany

## Abstract

To our knowledge, this is the first case of an arterial bleeding as a late complication 3 months after nephron sparing surgery of renal cell cancer, presumably originating from an arteriocalyceal fistula. Superselective embolization of the feeding arterial branch was chosen for treatment of the hemorrhage and proved successful. The high efficacy of superselective embolization as a minimally invasive procedure in this and other cases of bleeding Vessels should be the preferred method instead of open surgery.

## CASE REPORT

A 61 year old man was admitted with flank pain on the left side. Sonography revealed renal cysts, however one atypical cyst on the left side was highly suspicious of a tumor. Therefore, a CT scan was performed, which confirmed suspicion of a 3cm cystic renal carcinoma at the upper pole of the kidney. A nephron-sparing resection of pT1 N0 M0 G2 renal cell carcinoma was performed without any complications. During follow-up five years later the patient was admitted again with microhematuria and flank pain on the left side. Sonography and CT-scanning demonstrated a 4x5 cm cystic tumor at the lower pole of the left kidney (Fig. 1). The second nephron sparing surgery for a pT1 N0 M0 G1 renal cell carcinoma was performed without any complications. The pyelocalyceal system was opened during surgery but reconstructed thereafter. Before the first follow-up examination the patient presented two months later with a sudden onset of painless severe macrohematuria. Sonography showed no perineal or subcapsular hematoma or any other pathological condition. Cystoscopy showed bleeding from the left ureteral orifice. Selective renal arteriography was performed by percutaneous needle puncture and catheterization of the common femoral artery. An arterial blush from a lacerated vessel at the site of the tumor resection could be seen (Fig. 2). To embolize the bleeding vessel, a 5-F catheter was placed with its tip in the left femoral artery followed by coaxial placement of a 2.7-F flexible microcatheter (Tracker;

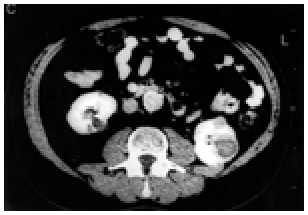

**FIGURE 1**. CT scan: Benign small cyst and cystic 4x5 cm renal cell carcinoma at the lower pole of the left kidney; TNM: pT1 N0 M0 G 1.

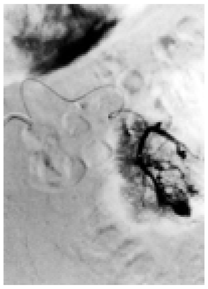

**FIGURE 2**. Pre-interventional angiogram demonstrates an arterial blush at the lower pole of the kidney at the site of former nephron sparing surgery.

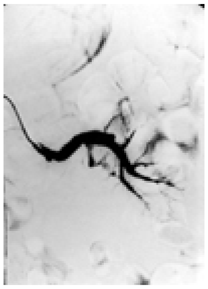

**FIGURE 3**. After selective transcatheter embolization, Digital Substraction Angiography demonstrates the occluded branch of the lacerated artery and disappearance of the arterial blush.

Target Therapeutics) which was brought into the periphery next to the lacerated artery. Embolization of the artery was performed using two microcoils (2 mm/10 mm microcoils; Target Therapeutics) Postembolization Digital Substraction Angiography demonstrated disappearance of the arterial blush (Fig. 3), the urine cleared, and the macrohematuria stopped 2 h afterwards. Post-interventionally the patient remained stable and was discharged the next day. After a follow-up of 3 months there was neither a tumor recurrence nor another episode of bleeding.

## DISCUSSION

Though radical nephrectomy is widely considered to be the treatment of choice for renal cell carcinoma, nephron sparing surgery or partial nephrectomy has emerged as a treatment option for unilateral, <4cm, low stage, localized renal cell cancer. Several studies were conducted to compare results of the radical and the organ preserving (tumor resection, partial nephrectomy) surgery. It has been shown that there was no significant difference between these two treatments regarding morbidity, mortality, or the recurrence rate [1-4]. However, nephron sparing surgery has the enormous advantage of preserving renal function without compromising cancer-free survival in most patients. Nephron sparing surgery is more challenging than radical nephrectomy, and therefore, can be more complicated. The complications after tumor resection are postoperative bleeding, macroscopic hematuria, urinary fistula, and acute renal failure requiring temporary dialysis [3]. Chronic renal insufficiency can be another rare late complication [5]. A review of the literature showed only few reports of patients with severe macroscopic hematuria after partial nephrectomy. Acute hemorrhagic complications were described directly after surgery and are usually due to insufficient perioperative control of small bleeding arteries. Interventional radiology with embolization of the feeding arterial branches for this condition can be successful [2]. Other possible treatments for bleeding after organ preserving surgery include open surgical revision or radical nephrectomy. To our knowledge, this is the first case of an arterial bleeding as a late complication 3 months after tumor enucleation of renal cell cancer. Cystoscopy localized the bleeding to the left upper urinary tract, presumably originating from an arteriocalyceal fistula. After arteriography revealed the bleeding site, superselective embolization of the feeding arterial branch was chosen for treatment of the hemorrhage and proved successful. The high efficacy of superselective embolization as a minimally invasive procedure in this and other cases of bleeding vessels led us to prefer this method instead of open surgery.
